# Neutrophil activation, acute lung injury and disease severity in *Plasmodium knowlesi* malaria

**DOI:** 10.1371/journal.pntd.0012424

**Published:** 2024-08-16

**Authors:** Angelica F. Tan, Sitti Saimah binti Sakam, Kim Piera, Giri S. Rajahram, Timothy William, Bridget E. Barber, Nicholas M. Anstey, Matthew J. Grigg, Steven Kho

**Affiliations:** 1 Global and Tropical Health Division, Menzies School of Health Research, Charles Darwin University, Darwin, Australia; 2 Infectious Diseases Society Kota Kinabalu Sabah – Menzies School of Health Research Clinical Research Unit, Kota Kinabalu, Malaysia; 3 QIMR Berghofer Medical Research Institute, Brisbane, Australia; 4 Clinical Research Centre, Queen Elizabeth Hospital, Kota Kinabalu, Malaysia; 5 Queen Elizabeth Hospital II, Ministry of Health Malaysia, Kota Kinabalu, Malaysia; 6 School of Medicine and Health Sciences, Monash University Malaysia, Kuala Lumpur, Malaysia; University of Florida, UNITED STATES OF AMERICA

## Abstract

The risk of severe malaria from the zoonotic parasite *Plasmodium knowlesi* approximates that from *P*. *falciparum*. In severe falciparum malaria, neutrophil activation contributes to inflammatory pathogenesis, including acute lung injury (ALI). The role of neutrophil activation in the pathogenesis of severe knowlesi malaria has not been examined. We evaluated 213 patients with *P*. *knowlesi* mono-infection (138 non-severe, 75 severe) and 49 *Plasmodium*-negative controls from Malaysia. Markers of neutrophil activation (soluble neutrophil elastase [NE], citrullinated histone [CitH3] and circulating neutrophil extracellular traps [NETs]) were quantified in peripheral blood by microscopy and immunoassays. Findings were correlated with malaria severity, ALI clinical criteria, biomarkers of parasite biomass, haemolysis, and endothelial activation. Neutrophil activation increased with disease severity, with median levels higher in severe than non-severe malaria and controls for NE (380[IQR:210–930]ng/mL, 236[139–448]ng/mL, 218[134–307]ng/mL, respectively) and CitH3 (8.72[IQR:3.0–23.1]ng/mL, 4.29[1.46–9.49]ng/mL, 1.53[0.6–2.59]ng/mL, respectively)[all p<0.01]. NETs were higher in severe malaria compared to controls (126/μL[IQR:49–323] vs 51[20–75]/μL, p<0.001). In non-severe malaria, neutrophil activation fell significantly upon discharge from hospital (p<0.03). In severe disease, NETs, NE, and CitH3 were correlated with parasitaemia, cell-free haemoglobin and angiopoietin-2 (all Pearson’s r>0.24, p<0.05). Plasma NE and angiopoietin-2 were higher in knowlesi patients with ALI than those without (p<0.008); neutrophilia was associated with an increased risk of ALI (aOR 3.27, p<0.01). In conclusion, neutrophil activation is increased in ALI and in proportion to disease severity in knowlesi malaria, is associated with endothelial activation, and may contribute to disease pathogenesis. Trials of adjunctive therapies to regulate neutrophil activation are warranted in severe knowlesi malaria.

## Author summary

The most common malaria in Malaysia is caused by animal-to-human transmission of *Plasmodium knowlesi* from its natural monkey hosts to humans via mosquito bites. As the human immune system responds to such infection, white blood cells such as neutrophils are recruited, activated and release products with pathogen-killing activity. There is increasing evidence that neutrophil products cause damage to the host and contribute to severe disease. While this has been studied in human-only malaria, the role of neutrophils in zoonotic *P*. *knowlesi* malaria is poorly understood. In this study, we examined three markers of neutrophil activation (neutrophil elastase, citrullinated histone H3 and neutrophil extracellular traps) in hospital patients with severe and non-severe knowlesi malaria from Sabah, East Malaysia. Compared to healthy controls, neutrophil activation was increased in patients with knowlesi malaria in proportion to disease severity. The increase in neutrophil activation was also linked to different types of severe malaria manifestations, especially acute lung injury. These findings have implications for development of new strategies to prevent host organ damage for better treatment of severe knowlesi malaria.

## Introduction

The monkey parasite *Plasmodium knowlesi* is the most common of eight emerging *Plasmodium* species causing zoonotic malaria [1]. Increasing zoonotic transmission of *P*. *knowlesi* impedes progress to malaria elimination [2]. From 2017 to 2021 Malaysia reported 17,125 *P*. *knowlesi* cases and 48 deaths, but no indigenous cases of *P*. *falciparum* or *P*. *vivax* [3]. The clinical spectrum of *P*. *knowlesi* infection extends from asymptomatic infection to severe disease [4,5], with the latter occurring in 6–9% of clinical cases, and 1 in 400 clinical cases leading to death [6]. Common clinical manifestations of severe knowlesi malaria include acute kidney injury, hyperparasitaemia, and respiratory distress [4,7]. Acute lung injury (ALI) and respiratory distress is a common cause of fatal outcome [6,8]. Underlying disease mechanisms in severe knowlesi malaria include impaired red blood cell (RBC) deformability [9], intravascular haemolysis from parasite-related RBC destruction [10], and endothelial activation mediated by increased osteoprotegerin (OPG) and angiopoietin-2 (Ang-2) levels released from Weibel-Palade bodies [11], all contributing to microvascular dysfunction, impaired perfusion and organ dysfunction [5,12,13].

In the presence of foreign pathogens, including *Plasmodium spp in vivo* [14,15], a cascade of innate responses in the host is triggered, including neutrophil activation and formation of neutrophil extracellular traps (NETs) [16]. NET formation (NETosis) involves neutrophilic degranulation [17], early post-translational modification of nuclear proteins such as citrullination of histone H3 (CitH3) [18], and subsequent extracellular release of deoxyribonucleic acid (DNA) and non-specific pro-inflammatory enzymes such as neutrophil elastase (NE) [19]. NE regulates the unique structure of NETs and has cell-killing activity through proteolytic mechanisms [20]. However, NETs induced by parasites [21], bacteria [22], fungi [23], and viruses [24] are also known to harm surrounding host environment, contributing to collateral tissue damage, likely exacerbated by persistence of infection.

Falciparum and knowlesi malaria have been associated with neutrophilia [4,7,25], and malaria pigment in neutrophils is frequently observed in severe falciparum malaria [26]. Malaria haemozoin has been associated with leucocyte activation through an inflammasome-dependent pathway [27], and respiratory distress in murine models [28]. Pulmonary neutrophil infiltrates were reported in ALI fatalities of falciparum [28] and vivax [29] malaria. Evidence of NET production has been demonstrated in the peripheral circulation of children with falciparum malaria [30], in adults with non-severe falciparum, malariae and vivax malaria [15], and is most prominent in adults with severe falciparum malaria [15,31]. Although NETs may inhibit *P*. *falciparum* growth in asymptomatic infections [15], elevated NETs and neutrophil activation in severe falciparum malaria are associated with tissue sequestration [31] and greater parasite biomass [15], suggesting a role for NETs in *P*. *falciparum* pathogenesis. In knowlesi malaria, peripheral parasitaemia is strongly associated with disease severity [4,7], and with markers of systemic inflammation and endothelial activation [13]. However, there remains limited understanding of the role of neutrophil activation products in the pathogenesis of clinical manifestation of severe malaria (SM), including ALI.

In the present study, we evaluated blood samples from patients with non-severe and severe *P*. *knowlesi* malaria to determine if neutrophil activation and NETosis is associated with host endothelial activation, ALI and severe disease.

## Methods

### Ethics statement

The studies were approved by the Medical Research and Ethics Committee of the Ministry of Health, Malaysia (NMRR-19-4109-52172 and NMRR-16-356-29088) and Menzies School of Health Research, Australia (HREC10-1431 and HREC16-2544). Written informed consent was obtained from all participating adults, or parent/guardian if aged<18 years.

### Study participants

The study was conducted between 2011 and 2022 in Sabah, Malaysia [3]. The following groups were enrolled: (1) non-severe malaria (NSM), defined as hospitalized with microscopy-diagnosed *P*. *knowlesi* infection, documented fever (>37.5°C) or history of fever in the last 48 hours, no clinical evidence of concurrent infection or major comorbidity, enrolled within 18 hours of commencing antimalarial treatment, and with no features of WHO-defined SM [32,33]; (2) SM, according to 2014 WHO clinical and laboratory research criteria, including acidosis (bicarbonate <15 mmol/L), acute kidney injury (creatinine >265 μM), anaemia (haemoglobin <7 g/dL in adults), jaundice (bilirubin >50 μM and parasitaemia >20,000/μL), shock, hyperparasitemia (>100,000 parasites/μL), and pulmonary oedema (radiologically confirmed, or recorded oxygen saturation of <92% on room air and respiratory rate >30 breaths/minute [32,33], and; (3) healthy controls (HC), with no fever in the preceding 14 days and negative blood smear for *Plasmodium* species. ALI was defined more broadly as those with recorded oxygen saturation of <94% [7] or requiring oxygen therapy at presentation, allowing inclusion of participants without an oxygen saturation or respiratory rate recorded on room air. Exclusion criteria included: (1) pregnant women, and (2) minors aged <5 years. Patients with *P*. *knowlesi* malaria received standard antimalarial treatment according to WHO guidelines [32]. Baseline clinical, laboratory, and demographic data were entered using standardized case report forms.

### Blood sample procedures

Peripheral venous blood samples were collected at baseline from all participants. Blood was collected again from a subset of patients during hospital care and before discharge. Hospital automated haematology and biochemistry laboratory results were recorded.

Thick and thin blood films were prepared from ethylenediaminetetraacetic acid (EDTA)-collected blood, and parasitaemia determined by a WHO-Level 1-equivalent research microscopist. Asexual parasitaemia was calculated per 200 white cells using microscopic parasite counts, and total white cell counts obtained from an automated analyser. Confirmation of *P*. *knowlesi* infection status and *Plasmodium* negativity in HCs were obtained by real-time PCR [34].

### Soluble markers of neutrophil activation, haemolysis and endothelial activation

Neutrophil activation markers NE and CitH3 were measured in heparinized plasma samples by enzyme-linked immunosorbent assay (ELISA), using Human Polymorphonuclear NE ELISA (Abcam, UK) and Human CitH3 ELISA (MyBioSource, USA) kits respectively. ELISAs were carried out per manufacturer’s instructions, with sample dilutions adjusted for each assay. CitH3 concentrations below limit of detection (1.2 ng/mL) were assigned a value of 0.6 ng/mL. Cell-free haemoglobin (CFHb), a marker of haemolysis, was measured in platelet-free plasma by ELISA (Bethyl Laboratories, TX). Endothelial activation markers Ang-2 and OPG were measured in heparinised plasma using quantikine ELISA kits and duoset ELISA, respectively (RnD Systems, USA).

### Microscopic identification of neutrophil extracellular traps

In Giemsa-stained thin smears, NETs were identified at 1000× magnification as extracellular structures with staining of decondensed chromatin in association with fragmented neutrophil-like cells or small granules, as validated previously [15]. A trained microscopist counted the number of NETs and neutrophils on each slide, with 10% of the slides counter-checked by a second experienced microscopist for concordance [15]. Slides with <50 neutrophils counted were excluded. NET counts were expressed as a percentage of neutrophils, then converted to NETs/μL blood using automated neutrophil counts. *P*. *knowlesi*-infected and uninfected RBCs observed in direct contact with NETs were also microscopically quantified. A small subset of patients had NETs and parasite counts measured at 6-hour intervals starting from initial blood sampling, then at 6, 12, 18, 24, and 48 hours after treatment.

Immunofluorescent staining of NETs was performed in a subset of patients on admission and at discharge from hospital to corroborate Giemsa-based NET microscopy. Thin smears were prepared from CTAD-anticoagulated blood on glass slides, dried and then stored at -80°C. Frozen slides were thawed, rapidly fixed and stained as described [15] using rabbit anti-human NE (Merck, Germany) and mouse anti-histone (clone H11–4 from Cell Death Detection ELISA kit, Roche, Germany), followed by secondary labelling with goat anti-rabbit IgG conjugated to fluorescein isothiocyanate and sheep anti-mouse IgG conjugated to cyanine-3 (Sigma Aldrich, USA). A Leica DMI8 confocal laser scanning microscope coupled to a Leica DC100 digital camera (Leica, Germany) was used to count NETs, identified as NE-positive cells with extracellular string-like structures of DNA positive for histones and 4′,6-diamidino-2-phenylindole (DAPI). Microscopy images were visualized and processed by Leica Application Suite X software (V3.7.5, Germany). Identical NETs parameters were calculated as per Giemsa-based microscopy.

### Statistical analysis

Analyses were performed using STATA v16 (TX, USA) and GraphPad Prism 7 (GraphPad Software, CA). SM, NSM, and HC group differences were tested using Kruskal-Wallis test for continuous variables, and Student *t*-test or the Wilcoxon rank-sum test for 2-group comparisons, including comparisons made using the same quantification methods against individual-level data on other malaria species from a previously published Indonesian dataset [15]. The Wilcoxon matched-pairs signed-rank test was applied to paired datasets (admission versus discharge). Geometric mean and exact binomial 95% confidence intervals (95%CI) for the distribution of parasite counts between SM and NSM groups were compared. Linear mixed-effects modelling was used to analyze 6-hourly time series Giemsa NET counts.

Associations between log-transformed NE, parasitaemia, and markers of disease severity were assessed using Pearson correlation. Linear regression evaluated the relationship between neutrophil activation and elevated inflammation markers in SM patients, adjusting for disease severity, age and parasitaemia. Spearman correlation was applied as a nonparametric measure of rank correlation between lowest oxygen saturation and plasma NE.

Logistic regression models were fitted to evaluate increasing plasma NE or neutrophil counts as predictors of ALI at the time of patient enrolment. To predict ALI outcome, logistic regression models were fitted, including controlling for age (≥45 years), neutrophilia (>7,700 neutrophils/μL using hospital automated counts) and log-transformed parasitaemia with backwards stepwise selection of variables method. Final model selection was evaluated using the area under the receiver-operating-characteristic curve (AUC).

## Results

### Patient baseline characteristics

Two-hundred-sixty-two study participants were enrolled between 2011–2022 from 6 districts across Sabah, comprising 75 severe and 138 non-severe patients with PCR-confirmed *P*. *knowlesi* mono-infections, and 49 PCR-negative controls (Table 1). Patients with knowlesi malaria had a higher median age and proportion of males than those in the HC group (p<0.001 respectively). Within the NSM group, male patients had a lower median age compared to female patients (36 vs 52 years respectively, p = 0.016).

**Table 1:** Baseline characteristics and clinical information of study participants.

Participant Characteristic	Healthy controls	*P*. *knowlesi*		*p-val* (NSM vs SM)	*p-val* (HC vs NSM)	*p-val* (HC vs SM)
Category		Non-severe	Severe			
Number (n)	49	138	75			
Age, years [IQR] (Range)	31 [26–39] (15–67)	39 [26–54] (10–85)	55 [45–64] (20–96)	<0.001	0.082	<0.001
Sex Male, n (%)	23 (47)	118 (84)	54 (74)	0.070	<0.001	0.002
^a^ History of chronic disease, n (%)	0	8 (17)	4 (22)	0.629	-	-
Previous self-reported malaria, n (%)	0	38 (36)	14 (35)	0.924	-	-
Fever duration, days (Range)	0	5 [3–7] (1–71)	5 [3–7] (1–50)	0.811	-	-
Parasitaemia, geometric mean (95% CI)	-	5,670 (4,230–7,600)	41,100 (26,600–63,500)	<0.001	-	-
White blood cell count (x1000/μL)	7.8 (6.7–8.8)	6.9 (5.8–8.2)	7.5 (5.7–10.4)	0.044	0.005	0.940
Neutrophils (x1000/μL)	4.14 (3.35–5.13)	4.10 (3.09–5.31)	4.97 (2.93–7.07)	0.030	0.549	0.137
Neutrophil (% of white cells)	55.2 (51.7–60.1)	60.8 (52.0–70.0)	65.6 (53.1–74.9)	0.052	0.013	<0.001
Hemoglobin count (g/dL)	13.8 (13.0–15.5)	13.3 (12.0–14.5)	12.3 (9.8–13.9)	0.001	0.018	<0.001
Red blood cell count (x1000/μL)	5.08 (4.88–5.51)	5.15 (4.68–5.53)	4.54 (3.57–5.22)	<0.001	0.871	0.004
Hematocrit (%)	40.2 (38.8–44.5)	40.4 (37.1–43.7)	37.1 (28.1–41.3)	<0.001	0.272	<0.001
Platelet count (x1000/μL)	327 (268–357)	67 (44–98)	42 (27–64)	<0.001	<0.001	<0.001
n/N Monocyte count (x1000/μL)	12/49 0.55 (0.39–0.91)	128/138 1.10 (0.75–1.45)	67/75 0.88 (0.53–1.35)	0.0613	0.006	0.119
n/N Neutrophil elastase concentration (ng/mL)	49/49 218 (134–307)	108/138 ^b^ 236 (139–448)	75/75 380 (210–930)	<0.001	0.303	<0.001
n/N Citrullinated histone concentration (ng/mL)	48/49 ^c^ 1.53 (0.60–2.59)	106/138 ^c^ 4.29 (1.46–9.49)	75/75 8.72 (3.00–23.1)	0.002	<0.001	<0.001
n/N Neutrophil extracellular traps (μL whole blood)	37/49 ^c^ 51 (20–75)	81/138 ^c^ 88 (38–181)	31/75 ^c^ 126 (49–323)	0.199	0.002	<0.001
n/N Cell-free haemoglobin (ng/mL)	33/49 ^c^ 36,300 (18,100–59,200)	29/138 ^c^ 39,700 (18,300–58,100)	71/75 ^c^ 71,700 (31,900–215,000)	0.007	0.772	0.001
Number of ALI on enrolment (n/N)	-	6/138	27/75	<0.001	-	-
n/N Angiopoietin-2 (pg/mL)	-	36/138 4,970 (3,500–8,530)	75/75 9,500 (5,350–15,200)	<0.001	-	-
n/N Osteoprotegerin (pg/mL)	-	30/138 2,260 (1,470–4,840)	74/75 3,260 (1,550–6,390)	0.204	-	-

Abbreviations: HC Healthy controls, NSM non-severe malaria, SM severe malaria, CI Confidence interval, ALI Acute lung injury

^a^ Patients presenting with non-malaria chronic disease, previously defined [4].

^b^ Excluded n = 30 NSM cohort 2 enrolled post-antimalarial treatment.

^c^ Excluded due to unsatisfactory sample quality or availability.

**Fig 1:**
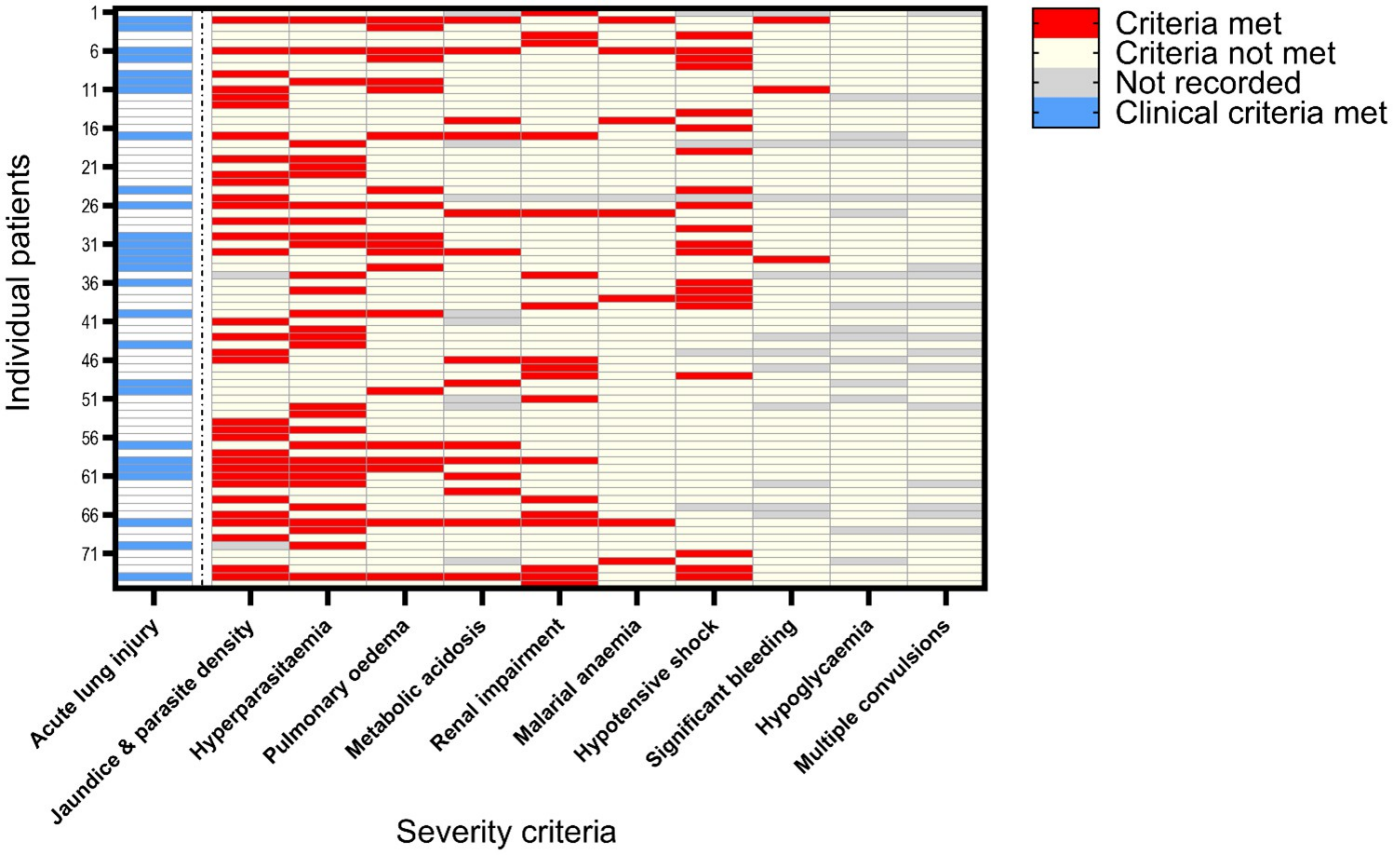
WHO-based severity criteria met by 75 SM, of which 27/75 met clinical criteria of acute lung injury (ALI) on enrolment.

The geometric mean parasitaemia of malaria patients was 11,200/μL (95%CI: 8,500–14,700/μL). SM patients had 7-fold higher geometric mean parasitaemia than NSM (p<0.001). Of the 75 adults with SM, 39 (52%) had a single WHO severity criterion and 36 (48%) met ≥2 criteria (Fig 1). The most common severe manifestation was jaundice, occurring in 44% (33/75) of SM patients, followed by 30 (40%) who met the hyperparasitaemia criterion of >100,000 parasites/μL. Of SM patients, WHO-defined pulmonary oedema occurred in 20 (27%). ALI was present in 33 (15%) knowlesi malaria cases overall, including in 27 (36%) with SM and 6 (4%) with NSM.

### Elevated neutrophil activation and peripheral NETs observed in knowlesi malaria patients

**Fig 2:**
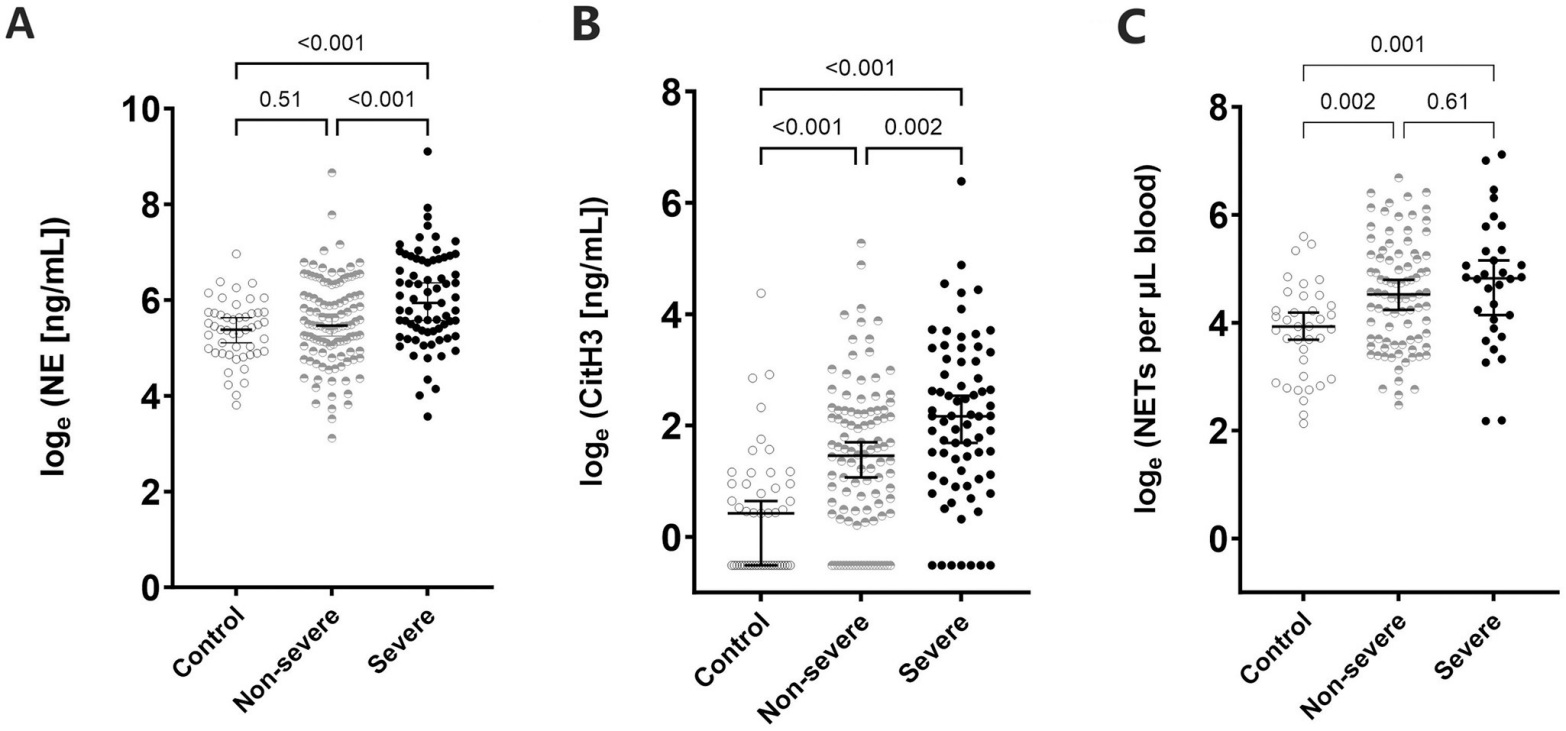
Plasma concentrations of neutrophil activation markers, (A) neutrophil elastase [NE], (B) citrullinated histone [CitH3], and (C) neutrophil extracellular traps [NET] counts in enrolled patients. Plots show individual data points compared via Student *t*-test, and median with 95% CI.

Plasma NE concentrations were elevated in SM (380 ng/mL [IQR:210–930 ng/mL]) compared to NSM (236 ng/mL [IQR:139–448], p<0.001) and HCs (218 ng/mL [IQR:134–307 ng/mL], p<0.001), Fig 2A). SM patients with ≥2 WHO severity criteria exhibited twice the plasma NE levels compared to those with a single criterion (median 566 vs 279 ng/mL, p = 0.045). Additionally, patients with jaundice had higher plasma NE levels compared to those without (median 633 vs 266 ng/mL, p<0.001).

Plasma CitH3 (early marker of NETosis) were available for 84% of patients. CitH3 concentration was highest in SM (8.72 ng/mL [IQR:3.00–23.1 ng/mL]) compared to NSM (4.29 ng/mL [IQR:1.46–9.49 ng/mL], p = 0.002) and controls (1.53 ng/mL [IQR:0.60–2.59], p<0.001, Fig 2B).

NETs were assessed in 149 Giemsa-stained slides: 31 SM, 81 NSM and 37 HCs. The median number of NETs/μL blood was elevated in SM (126/μL [IQR:49–323/μL]) and NSM (88/μL [IQR:38–181/μL]) compared to controls (51/μL [IQR:20–75/μL], p≤0.002 for both), although the difference between SM and NSM groups were not significant (Fig 2C).

There were consistent positive correlations between each of the markers of neutrophil activation (NE, NETs and CitH3) observed in SM (all correlations r>0.474, p<0.007), including after controlling for parasitaemia and age (S1 Fig). In NSM, positive correlations were observed between NE and both NETs and CitH3 (r>0.245, p<0.028) but not between CitH3 and NETs.

### P. knowlesi-infected RBCs (iRBCs) in direct contact with NETs

**Fig 3:**
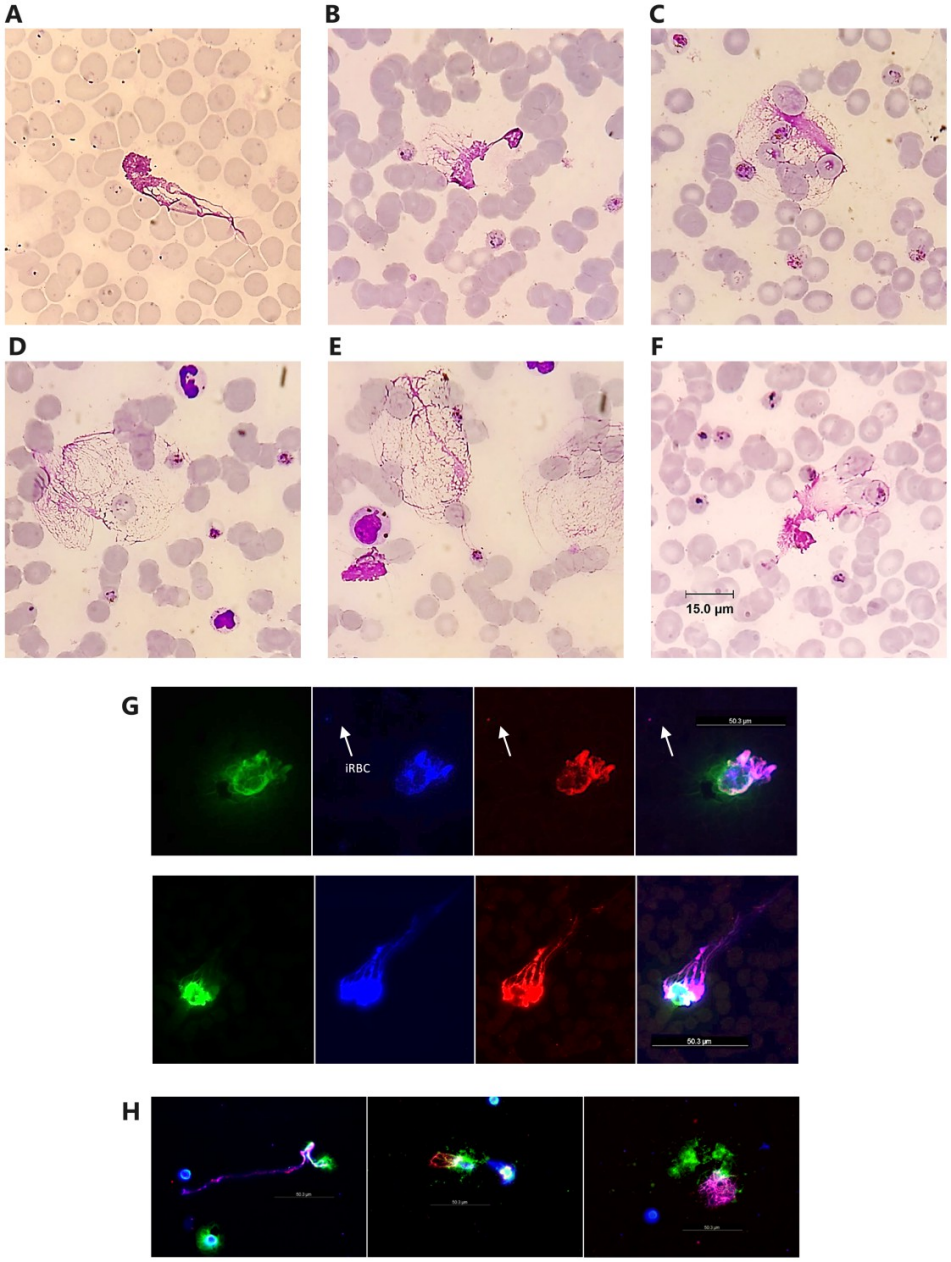
Circulating NETs identified in knowlesi malaria by Giemsa stain, with parasitized red blood cells (iRBCs) (scale bar in 3F applies to 3A – 3E). 3G. 3-colour immunofluorescent-stained (IF) NETs structures, positive for NE, deoxyribonucleic acid (DNA), and histones (left-to-right), and (3H) corresponding overlay images (scale bar in 3G also applies to 3H).

All knowlesi malaria patients had iRBCs in direct contact with NETs, with a higher proportion found in SM than in NSM (56% vs 31% of NETs respectively, p = 0.007, Fig 3A–3F).

The median number of NETs containing iRBCs was 1 (IQR:1–3; upper range 10) in SM and 1 (IQR:1–2; upper range 6) in the NSM group. The majority of NET-bound iRBCs in the SM group consisted of 1 to 2 late trophozoite stages, with other stages seen less frequently. The highest number of iRBCs present in a single NET was 4, consisting of 3 late trophozoites and 1 schizont. All NSM patients with NET-bound iRBCs had a single asexual-stage parasite (ring, trophozoite or schizont) present. NETs in the SM group were more frequently in contact with uninfected RBCs compared to the NSM group (median 12 [IQR:7–14] vs 8 [IQR:5–11], respectively, p = 0.011).

Slides examined by immunofluorescent microscopy showed NET-like structures with co-staining of NE, histones and nuclear material (DAPI), confirming the presence of extracellular traps of neutrophil origin in *P*. *knowlesi* malaria (Fig 3G). Although the immunofluorescent assay could identify iRBCs in close proximity to NETs, parasites were not quantified inside immunofluorescent NETs due to overlap in the staining of parasite and host nuclear material (Fig 3H). NETs counts evaluated by both Giemsa and IF microscopy approaches were within 95% limit of agreement on Bland-Altman testing, with the exception of a single outlier (S2 Fig).

### Neutrophil activation and associations with parasitaemia, intravascular haemolysis and endothelial activation

**Fig 4:**
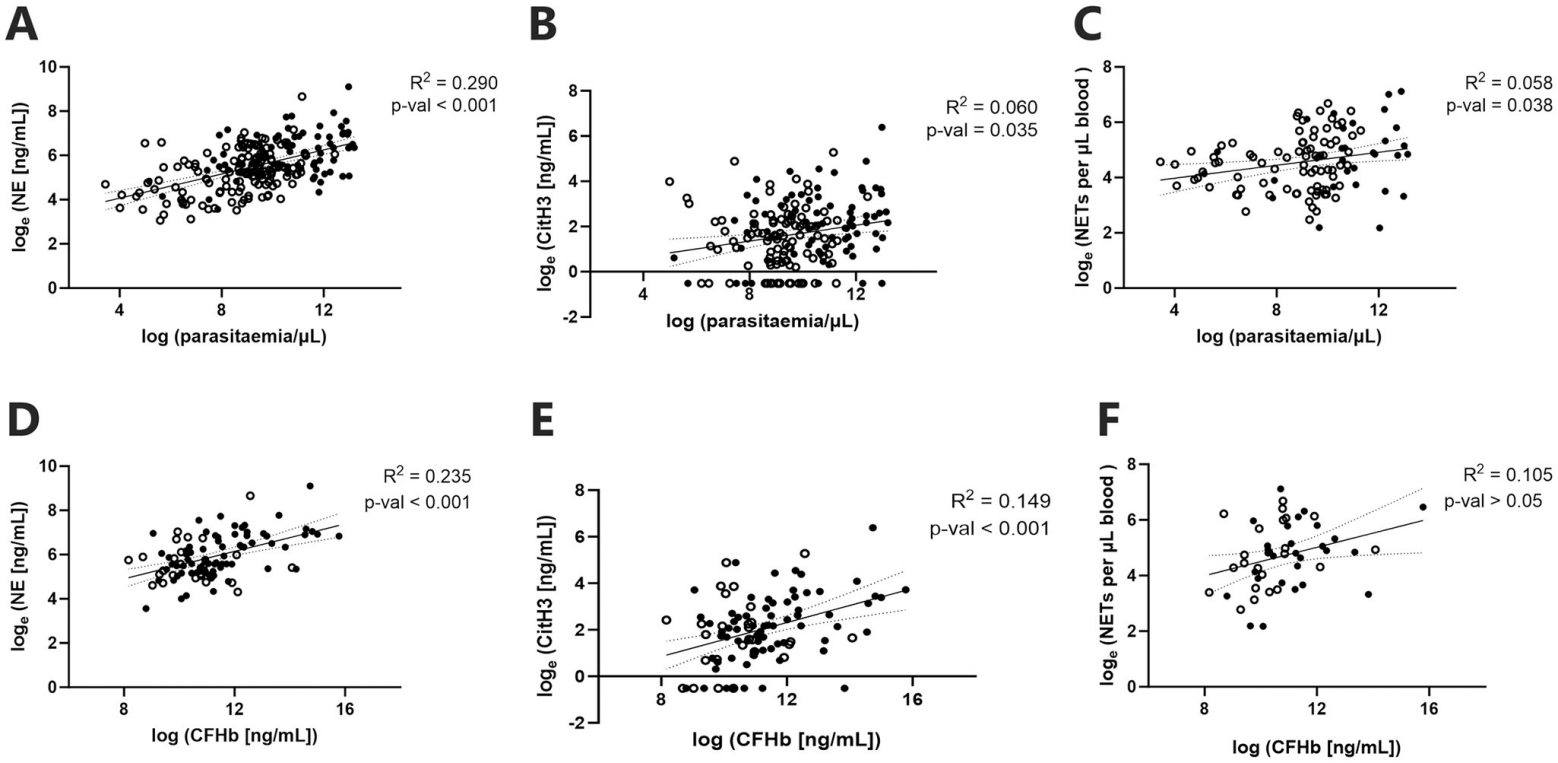
Correlations of (A-C) parasitaemia and (D-F) cell-free haemoglobin (CFHb) with (A and D) NE, (B and E) CitH3, and (C and F) Giemsa-based NET counts. Linear regression applied for knowlesi cases with disease severity as covariate (indicated by solid line with 95% CI dotted-line, filled-circles indicate SM and unfilled-circles indicate NSM).

Plasma NE concentrations correlated with increasing parasitaemia in both SM and NSM groups (Pearson r>0.413, p<0.001), Fig 4A. These findings remained significant after controlling for age, neutrophil count and disease severity (R^2^ = 0.328, p<0.001, univariate associations detailed in S1 Table). Parasite counts for SM patients also correlated with CitH3 (Pearson r = 0.240, p = 0.035, Fig 4B), while peripheral NET counts controlling for disease severity showed a positive correlation (R^2^ = 0.058, p = 0.038, Fig 4C).

Plasma CFHb concentrations were elevated in SM compared to NSM (p = 0.007) and controls (p = 0.001), with no difference observed between NSM and HC (Table 1). CFHb was found to correlate with NE and CitH3 in SM (Pearson r>0.442, p<0.001), but not with NET counts (Fig 4D–4F). No correlation was observed between all three markers of NETosis and CFHb in the NSM group. A fitted model controlling for age, neutrophil count and disease severity determined that plasma NE levels increase by 68.6% for each unit increase in CFHb (ng/mL) and parasite/μL (R^2^ = 0.335, p<0.001).

**Fig 5:**
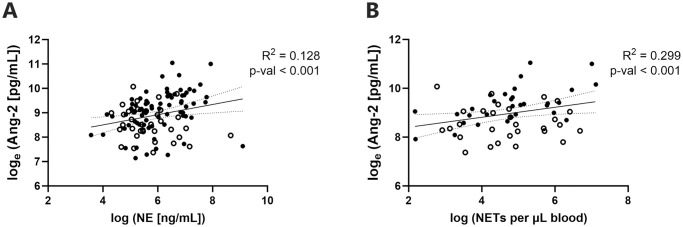
Relationship between endothelial activation marker angiopoetin-2 (Ang-2) and (A) NE and (B) NETs. Linear regression applied for knowlesi cases with disease severity as covariate (indicated by solid line with 95% CI dotted-line, filled-circles indicate SM and unfilled-circles indicate NSM).

Plasma concentrations of Ang-2 were twice as high in SM compared to NSM (9500 ng/mL vs 4970 ng/mL, p = 0.002), while OPG levels were similar between these groups. A positive correlation was apparent between both NE and NETs with Ang-2 in SM patients (Pearson r>0.363, p<0.001, Fig 5A–5B), but not in the NSM group. Linear regression controlling for age and parasitaemia in the SM group indicated an increase in Ang-2 by 0.5 pg/mL for every 1ng/mL increment of NE (R^2^ = 0.335, p = 0.019). Similarly in SM, linear regression performed controlling for age and parasitaemia determined a two-fold increase in both NET and parasite counts results in a 35.2% increase in Ang-2 (R^2^ = 0.478, p<0.001).

### Neutrophil activation in ALI

**Fig 6:**
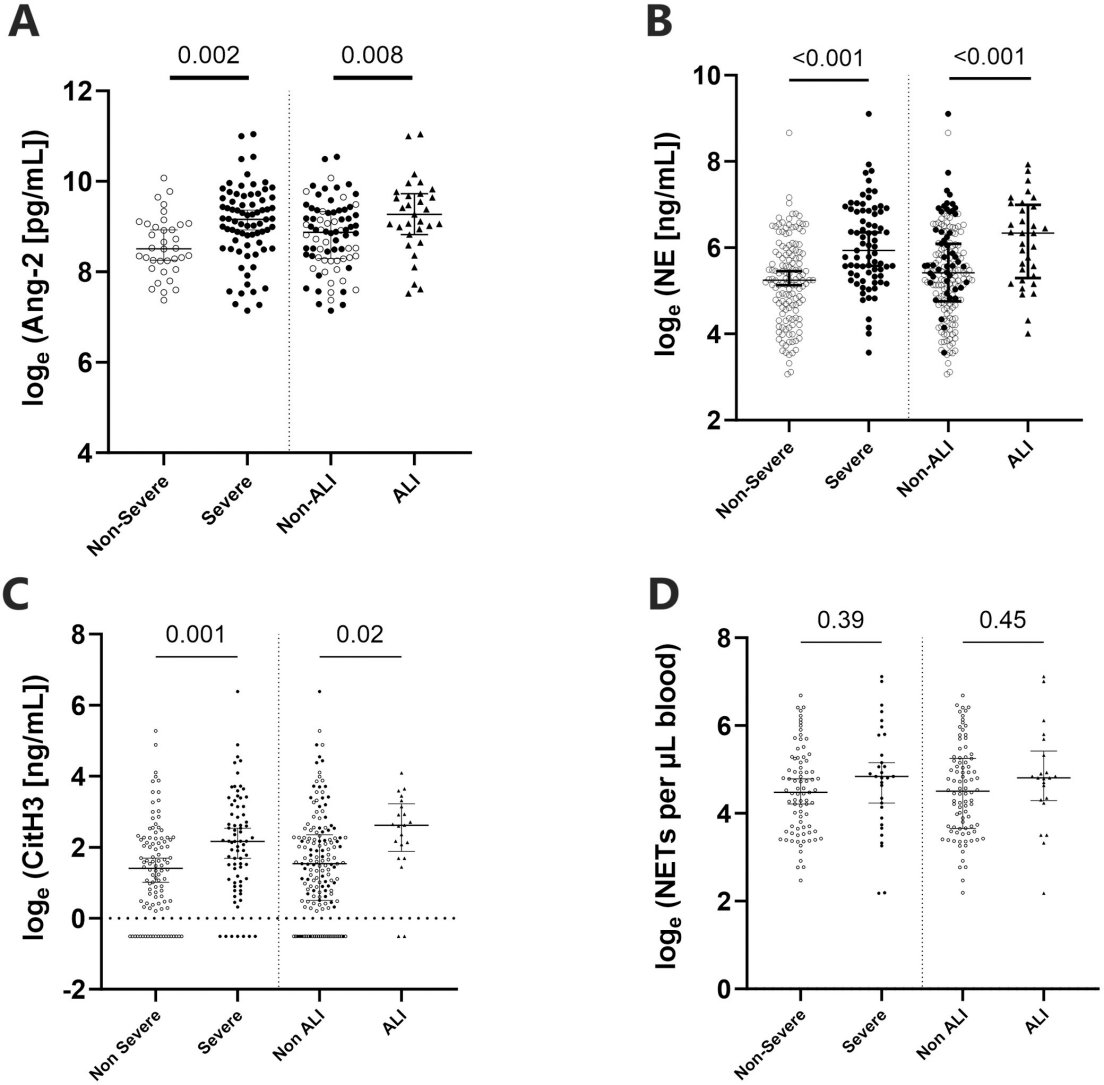
Plasma concentrations of (A) Ang-2, (B) NE, (C) CitH3, and (D) NETs in relation to SM and ALI. Plots show individual log_e_-transformed data points compared via Student t-test, and median with 95% CI.

There were 33 *P*. *knowlesi* cases with ALI at presentation. Patients who met criteria for ALI compared to non-ALI exhibited higher plasma concentrations of Ang-2 (10,600 ng/mL vs 7,120 ng/mL, p = 0.008, Fig 6A). Total neutrophil count was higher in those with ALI (median 5.7 vs 4.0 x10^3^/μL; p<0.001), including a higher proportion with neutrophilia (48% vs 16%; p<0.001). Plasma NE concentrations were two-fold higher than those without ALI (median 565 vs 225 ng/mL; p<0.001, Fig 6B). Similar to findings with NE, plasma CitH3 was higher in those with ALI (median 11.4 vs 4.7 ng/mL; p = 0.02, Fig 6C). However, there was no difference in peripheral NET counts between the two groups (median 122/μL [n = 22/33] vs 90/μL; p = 0.45, Fig 6D).

There was an inverse correlation between plasma NE levels and patient oxygen saturation levels at presentation (Spearman’s rho = -0.191, p = 0.009). Although univariable logistic regression showed that NE was a predictor variable for ALI (aOR 2.00, p<0.001), it was not an independent predictor when controlling for parasitaemia, age and neutrophil counts on multivariable logistic regression (Model 1, S2 Table). However, high neutrophil counts proved to be an independent predictor of ALI (aOR 3.22, 95%CI: 1.52–6.82, p<0.005, Model 1). The model demonstrated an AUC of 0.817 (95%CI: 0.738–0.896).

In a second multivariate logistic regression model for clinical predictors of ALI at hospital presentation with routinely collected demographic and laboratory data, neutrophilia (aOR 4.30, 95%CI:1.42–13.0, p = 0.01) and age>45 years (aOR 3.41, 95%CI:1.34–8.68, p = 0.010) remained independent clinical predictors of ALI when controlling for the other known major association with severe disease (parasite count>15,000/μL). The model demonstrated an AUC of 0.75 (95%CI 0.65–0.89) for ALI detection (Model 2, S2 Table).

### Neutrophil activation is reduced after antimalarial treatment

A subset of 39 NSM patients had paired samples collected on day of enrolment and on discharge from hospital. Median time to discharge was 3 days (IQR:3–4 days). On day of enrolment, these patients were sampled a median of 3 hours (IQR:1–6 hours) after antimalarial administration.

**Fig 7:**
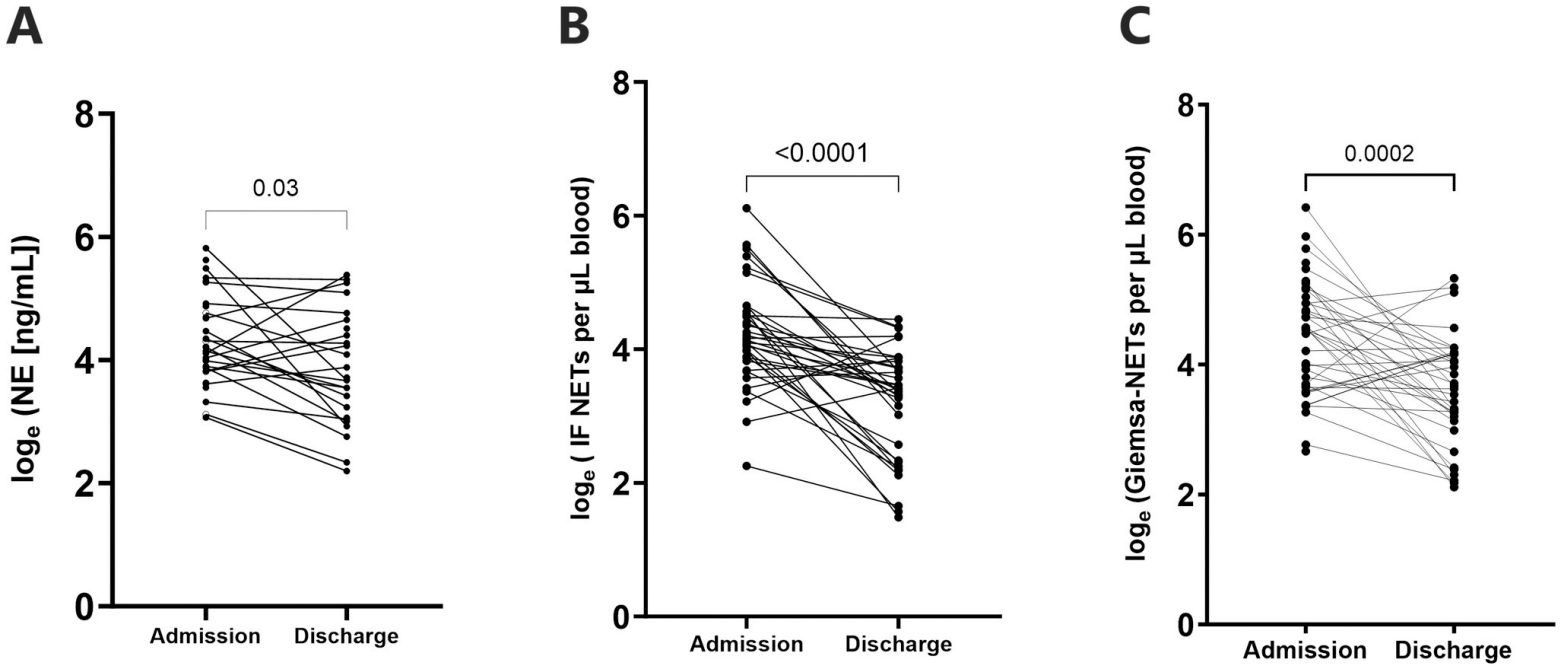
Neutrophil activation of knowlesi malaria inpatients on admission and discharge from hospital. Paired data available for (A) NE, and NETs using both (B) IF and (C) Giemsa-microscopy methods.

Plasma NE concentration and NETs counts were lower at discharge compared to admission. Plasma NE reduced from a median 63 to 40 ng/mL (p = 0.034, Fig 7A). NETs counts derived by IF decreased from a median of 59 to 30/μL, and for Giemsa from 100 to 39/μL (p<0.001 for both, Fig 7B and 7C respectively).

In the 13 patients with serial 6-hourly post-treatment measurements, a linear mixed effects model demonstrated that NET counts decreased by an average of one NET per hour (B = -0.026, p<0.001) from baseline NET counts (Wald χ^2^ = 21.65, p<0.001; S3A Fig). This is consistent with the observed decrease in parasite counts after administration of anti-malarial treatment (S3B Fig).

## Discussion

Our study demonstrates neutrophil activation occurring in proportion to *P*. *knowlesi* disease severity, as evidenced by proportional increase in peripheral NET formation, and the plasma markers NE and CitH3. NE, CitH3 and plasma Ang-2 were elevated in patients with ALI, suggesting a role for neutrophil activation in the pathogenesis of ALI in knowlesi malaria. Neutrophil activation in knowlesi malaria patients resolved upon completion of antimalarial treatment, with circulating NETs and plasma NE levels returning to the levels of healthy controls, consistent with rapid resolution of parasite-induced NETosis after parasite clearance. Immunofluorescent NET counts using gold-standard reference markers (NE and histones) highly corroborated with Giemsa-stained microscopy quantification, supporting that the network of decondensed chromatin structures observed under light microscopy were of neutrophil origin. Additionally, plasma NE and CitH3 concentrations were determined to have positive correlation with both microscopic methods of NET quantification.

High neutrophil counts, including those meeting the clinical threshold for neutrophilia, have been previously associated with severe knowlesi malaria [7,13,35], and reaffirmed in the current study. High neutrophil granule-related gene expression has also been linked to high parasite biomass in severe falciparum [36] and vivax malaria [37].

Compared to published Indonesian datasets in *P*. *falciparum* SM [15], peripheral NETs were lower in our *P*. *knowlesi* SM cases (S4 Fig), potentially a reflection of the lower parasitemia in *P*. *knowlesi* SM cases compared to *P*. *falciparum* SM in the Indonesian cohort. However, in contrast to NETs, plasma NE levels between the two species were similar in SM [15], suggesting that similar levels of systemic neutrophil activation may be associated with subsequent microvascular and tissue inflammation in severe disease by both *Plasmodium* species. However, the spectrum of organ dysfunction in these two geographically-distinct patient populations are different, thus limiting our interpretation. Comparisons of NE and NETs between published NSM patients [15] and our knowlesi cases revealed that *P*. *knowlesi* had higher concentrations of plasma NE than *P*. *vivax* and *P*. *malariae* (S4 Fig), despite similar parasite counts, suggesting a comparatively higher inflammatory systemic response [5] and associated NETosis compared to other non-falciparum species.

In severe knowlesi patients, peripheral parasitaemia and intravascular haemolysis were independently associated with plasma NE levels, suggesting both contribute to neutrophil activation in severe disease. This is consistent with *in vivo* data [31] demonstrating that plasma from SM, but not NSM, was able to induce NETosis in healthy neutrophils. NETosis and formation of intravascular NETs are triggered by proinflammatory cytokines [31], such as tumor necrosis factor–α and interleukin-1β. Production of these molecules are, in turn, driven by extracellular heme released from iRBCs when parasites egress. Despite the presence of iRBCs within NETs suggesting an antiparasitic role in *P*. *knowlesi* infections, the collective cytotoxic oxidative effects on host vascular endothelium likely contribute to pathological inflammatory responses leading to severe disease [38].

In severe knowlesi malaria, elevated concentrations of plasma Ang-2 in the presence of increased parasite biomass indicate systemic inflammation and endothelial activation [12], which may lead to microvascular dysfunction similar to that observed in severe falciparum malaria [39–41]. Consistent with previous reports [11], increased plasma levels of Ang-2 were observed in proportion to disease severity. In the SM cohort, plasma NE was correlated with Ang-2 independent of parasitaemia, consistent with a previous study reporting an association between neutrophilia and Ang-2 in severe knowlesi malaria [12].

Although data on the relationship between Ang-2 and NE in malaria is limited, with only one study to date described a positive correlation between NE and Ang-2 in a murine spinal injury model [42], other studies have demonstrated that upregulation of Ang-2 can enhance neutrophil migration [43] and induce NETosis under *in vitro* [44] and *in vivo* [45] conditions. It is plausible that this process may exhibit bi-directionality.

Activated neutrophil products modulate endothelial cell receptors and coagulation processes, promote adherence of iRBCs and increase microvascular permeability in falciparum malaria [31]. As previously reported in severe *P*. *falciparum* and *P*. *vivax* malaria [15,31], NETosis has potential to contribute to organ dysfunction [12] via endothelial inflammatory pathways [31]. In falciparum malaria, retinopathy-positive cerebral malaria is specifically associated with vascular accumulation of externalized neutrophil proteins such as NE and proteinase-3 [46].

In this study, higher concentrations of plasma Ang-2,NE and CitH3 in patients with ALI support previously described association between neutrophilia and parasite-induced lung inflammation [7]. Our study did not find peripheral NETs occurring more frequently in knowlesi malaria-associated ALI, unlike the increase in lung tissue NETs in experimentally-induced pulmonary complications of mice infected with *P*. *berghei* [47]. We were unable to sample lung tissue NETs in our human studies. Nevertheless, the association between ALI and plasma NE, a measure of total body neutrophil activation, is consistent with a role of neutrophil activation in the pathogenesis of ALI in knowlesi malaria.

In addition to pigmented neutrophils, the presence of malarial pigment in monocytes is associated with disease severity in falciparum malaria [48,49]. Pigmented neutrophils and monocytes have also been observed in knowlesi malaria [50,51]. Markedly increased pulmonary phagocytic activity, likely from both neutrophils and mononuclear phagocytes, is seen in uncomplicated falciparum and vivax malaria [52]. In post-mortem studies of falciparum malaria, accumulation of monocytes in pulmonary vessels [53] suggest a contribution to ALI that may occur in tandem with neutrophils through IL-33-mediated pathways [54].

Our study had several limitations. We measured circulating NETs in peripheral blood but were unable to quantify microvascular and tissue NETs. Nevertheless, measures of systemic neutrophil activation accounted for both circulating and non-circulating neutrophil activation. Although a smaller proportion of peripheral NETs were quantifiable using immunofluorescent microscopy, findings were supported by Giemsa-based analysis in a larger sample size, enabling confirmation that NETs increase with disease severity. We used peripheral parasitemia as a measure of total parasite biomass for associations with NE and NETs counts and disease severity, rather than plasma markers of total biomass. Although *P*. *knowlesi* iRBCs can accumulate in microvasculature of organs [51], peripheral parasitaemia is a robust independent predictor of *P*. *knowlesi* disease severity [4,13]. Incorporation of additional neutrophil activation markers, e.g., myeloperoxidase [20], lysozyme and neutrophil-specific granule proteins [55], and pro-inflammatory chemokines [56] may further elucidate involvement of neutrophil activation in knowlesi malaria pathogenesis. There is overlap of severe malaria syndromes in severe knowlesi malaria, however despite the relatively large sample size for severe knowlesi malaria series, we did not have the power to examine the independence of relationships for most individual severe malaria syndromes. The absence of deaths in our series, precluded examination of relationships with outcomes. Our study design did not allow for delineation of a causative relationship. Nevertheless, biological associations found in our study suggest a role of neutrophil activation in knowlesi malaria pathogenesis. The results of our analyses support the notion that neutrophil activation is exacerbated by higher parasitaemia, increased haemolysis, and endothelial activation.

In conclusion, neutrophil activation and circulating NET formation increased in proportion to clinical severity in *P*. *knowlesi* malaria. Associations with parasite counts and endothelial activation support the notion of neutrophil activation contributing to knowlesi malaria pathogenesis. Further evaluation of adjunctive therapies to modulate neutrophil activation and NETosis in vascular infections are recommended.

## Supporting information

S1 Table Univariate analysis for all three markers of neutrophil activation against relevant clinical parameters. *Spearman correlation coefficient reported for non-parametric variables.
(PDF)

S2 Table Multivariable logistic regression of clinical predictors for acute lung injury in knowlesi malaria and model comparisons*, (*n* = 198). Variables parasitaemia, neutrophil elastase, cell-free haemoglobin and angiopoietin-2 are log-transformed, while neutrophil count and age are square-root-transformed.
(PDF)

S1 Fig Linear regression conducted between NE, CitH3 and NET counts (Giemsa) of SM group, controlling for age and parasitaemia. (TIF)

S2 Fig **(A)** Bland-Altman method-comparison plot between Giemsa and immunofluorescent (IF) NET counts, dotted lines denote within 95% limit of agreeability. **(B)** Giemsa and IF NET counts of discharged patients return to control (HC) levels as indicated by non-significant p-values of >0.05 (ns).
(TIF)

S3 Fig **(A)** Subset of 13 non-severe individuals with 6-hour serial measurements of Giemsa-based NET counts, and **(B)** corresponding ratio of decreasing parasitaemia with decreasing NETs.
(TIF)

S4 Fig Comparison of **(A)** neutrophil elastase, **(B)** circulating neutrophil extracellular traps and **(C)** parasitaemia of *P*. *knowlesi* against *P*. *falciparum*, *P*. *vivax* and *P*. *malariae* (from Timika, Indonesia [15]) in severe and non-severe cohorts.
(TIF)

S1 Dataset Deidentified dataset that was utilized in the analysis. (XLS)
