# Supplementary material for: Neutrophil activation, acute lung injury and disease severity in Plasmodium knowlesi malaria
Source: PLoS Negl Trop Dis. 2024 Aug 16;18(8):e0012424. doi: 10.1371/journal.pntd.0012424 (PMC11357107; doi:10.1371/journal.pntd.0012424)
Supplement: S1 Table — *Spearman correlation coefficient reported for non-parametric variables. (PDF) [file pntd.0012424.s001.pdf]

Supplementary Table 1. Univariate analysis for all three markers of neutrophil activation against relevant clinical parameters. *Spearman correlation coefficient reported for non-parametric variables.

	Neutrophil elastase (ng/mL)		NET counts (µL)		Citrullinated histone (ng/mL)	
	**r**	**p-value**	**r**	**p-value**	**r**	**p-value1**
Disease severity (Categorical)	0.363	<0.001	0.115	0.228	0.252	<0.001
Age (years)	0.175	0.011	0.098	0.306	0.208	0.053
Parasitaemia (µL)	0.522	<0.001	0.241	0.011	0.210	0.005
Neutrophils (x1000/µL)	0.331	<0.001	0.449	<0.001	0.111	0.156
Citrullinated histone (ng/mL)	0.572	<0.001	0.193	0.014		
NETs per µL blood	0.332	0.002			0.193	0.014
Cell-free haemoglobin (ng/mL)	0.484	<0.001	0.309	0.028	0.385	<0.001
Angiopoeitin-2 (pg/mL)	0.253	0.007	0.300	0.020	0.375	0.024
Osteoprotegerin (pg/mL)	0.098	0.323	0.209	0.126	0.185	0.052
Oxygen saturation*	-0.191	0.009	-0.139	0.145	-0.121	0.134
Respiratory rate*	0.221	0.002	0.084	0.378	0.062	0.445
