# Supplementary material for: Neutrophil activation, acute lung injury and disease severity in Plasmodium knowlesi malaria
Source: PLoS Negl Trop Dis. 2024 Aug 16;18(8):e0012424. doi: 10.1371/journal.pntd.0012424 (PMC11357107; doi:10.1371/journal.pntd.0012424)
Supplement: S2 Table — Variables parasitaemia, neutrophil elastase, cell-free haemoglobin and angiopoietin-2 are log-transformed, while neutrophil count and age are square-root-transformed. (PDF) [file pntd.0012424.s002.pdf]

Supplementary Table 2: Multivariable logistic regression of clinical predictors for acute lung injury in knowlesi malaria and model comparisons*, (*n* = 198).

Parameters	Crude OR	95% CI		p-val	Model 1 aOR	95% CI		p-val	Model 2 aOR	95% CI		p-val
		Lower	Upper			Lower	Upper			Lower	Upper	
					LR chi^2: 38.4			<0.001	LR chi^2: 22.4			<0.001
Age (years)	1.5	1.1	2.1	0.010	1	1.0	1.1	0.033	-	-	-	-
Age (>45 years)	3.7	1.6	8.5	0.003	-	-	-	-	3.4	1.3	8.7	0.01
Parasitaemia (µL)	1.8	1.4	2.3	<0.001	1.4	1	1.9	0.049	-	-	-	-
Parasite count >15,000/µL	3.1	1.4	6.7	0.005	-	-	-	-	2	0.8	4.9	0.153
Neutrophils (x1000/µL)	4.8	2.3	9.7	<0.001	3.3	1.5	7	0.002	-	-	-	-
Neutrophilia (>7700/µL)	6.5	2.4	17.5	<0.001	-	-	-	-	4.3	1.4	12.9	0.01
Neutrophil elastase (ng/mL)	2.0	1.4	2.9	<0.001	0.9	0.6	1.7	0.99				
Cell-free haemoglobin (ng/mL)	1.4	1.0	1.9	0.023	-	-	-	-	-	-	-	-
Citrullinated histone (ng/mL)	1.4	1.0	1.8	0.023	-	-	-	-	-	-	-	-
Angiopoeitin-2 (pg/mL)	2.1	1.1	3.8	0.011	-	-	-	-	-	-	-	-
**Area under ROC curve**				0.013*	0.82	0.74	0.89		0.75	0.65	0.89	
**Sensitivity**					3.5	0.1	17.8		24.1	10.3	43.5	
**Specificity**					100	97.8	100		97.0	93.2	99.0	
**Positive predictive value**					100	2.5	100		58.3	27.7	84.8	
**Negative predictive value**					85.8	80.1	90.3		88.2	82.6	92.4	
**Correctly classified %**					87.9				86.4			
