# Supplementary figures and images for: Neutrophil activation, acute lung injury and disease severity in Plasmodium knowlesi malaria
Source: PLoS Negl Trop Dis. 2024 Aug 16;18(8):e0012424. doi: 10.1371/journal.pntd.0012424 (PMC11357107; doi:10.1371/journal.pntd.0012424)

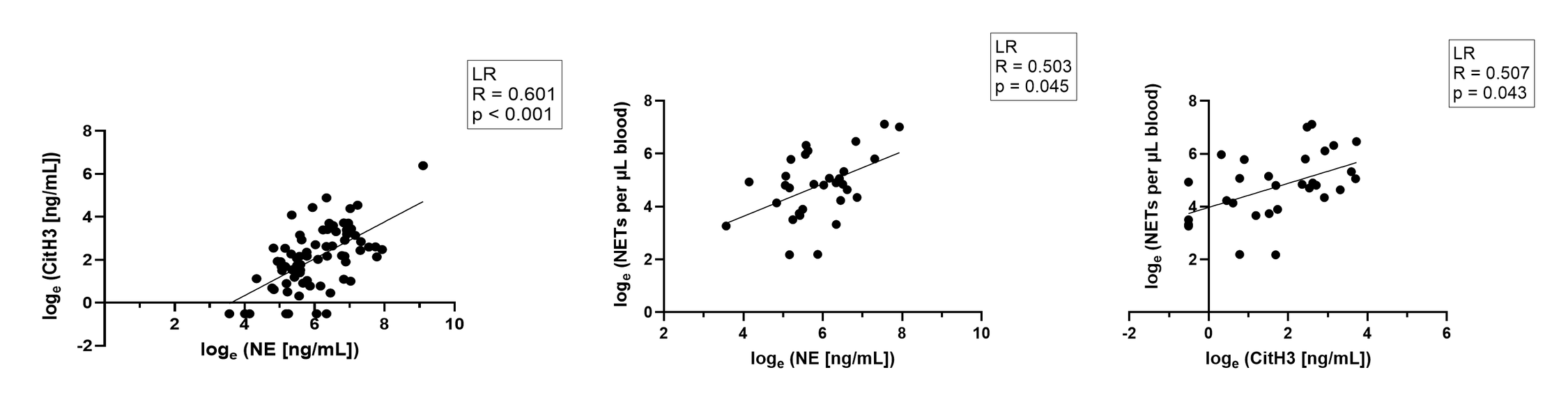

Supplement: S1 Fig — (TIF) [file pntd.0012424.s003.tif]

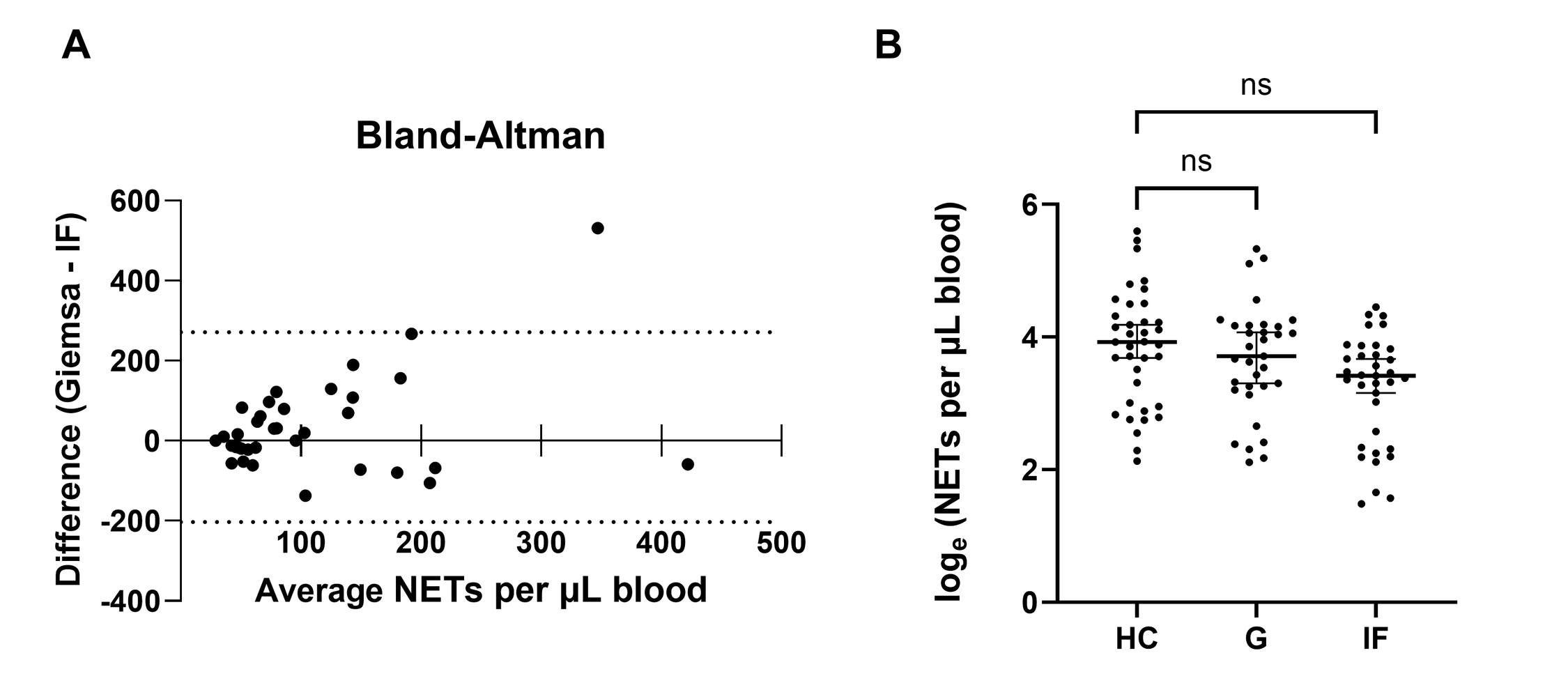

Supplement: S2 Fig — (A) Bland-Altman method-comparison plot between Giemsa and immunofluorescent (IF) NET counts, dotted lines denote within 95% limit of agreeability. (B) Giemsa and IF NET counts of discharged patients return to control (HC) levels as indicated by non-significant p-values of >0.05 (ns). (TIF) [file pntd.0012424.s004.tif]

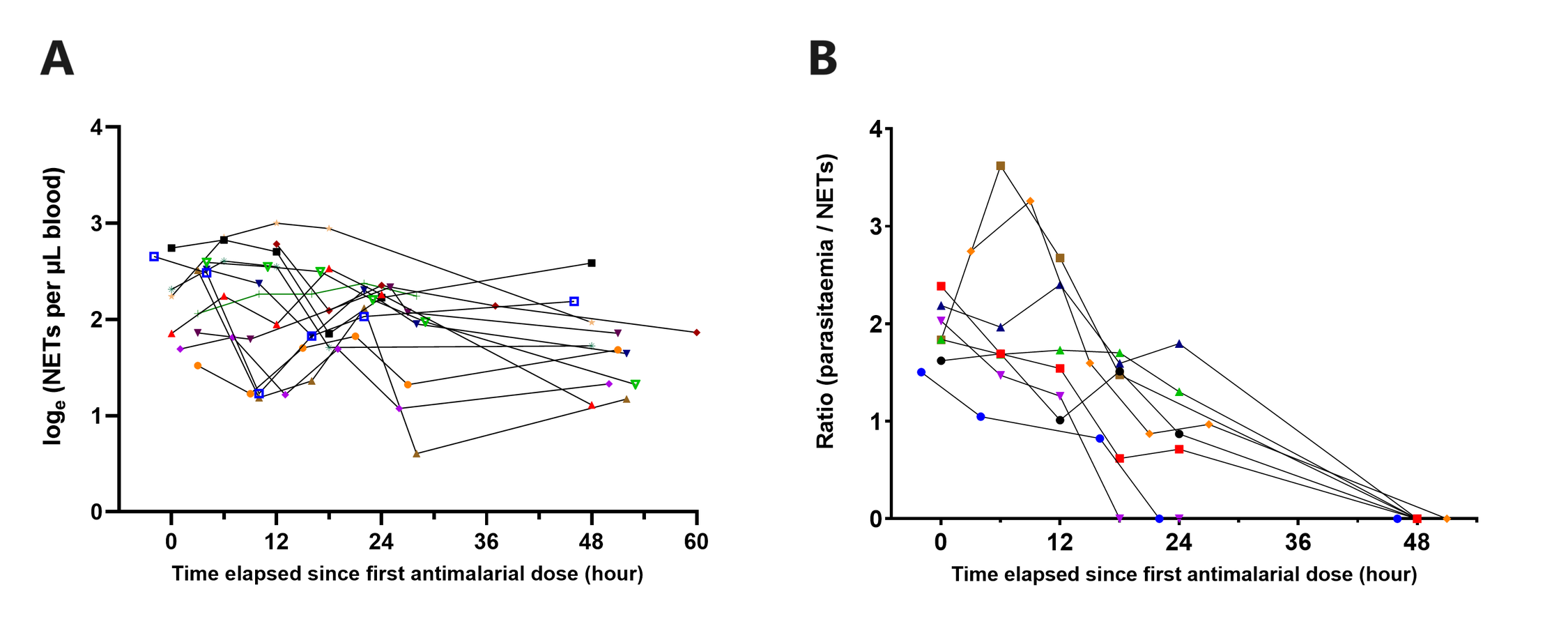

Supplement: S3 Fig — (A) Subset of 13 non-severe individuals with 6-hour serial measurements of Giemsa-based NET counts, and (B) corresponding ratio of decreasing parasitaemia with decreasing NETs. (TIF) [file pntd.0012424.s005.tif]

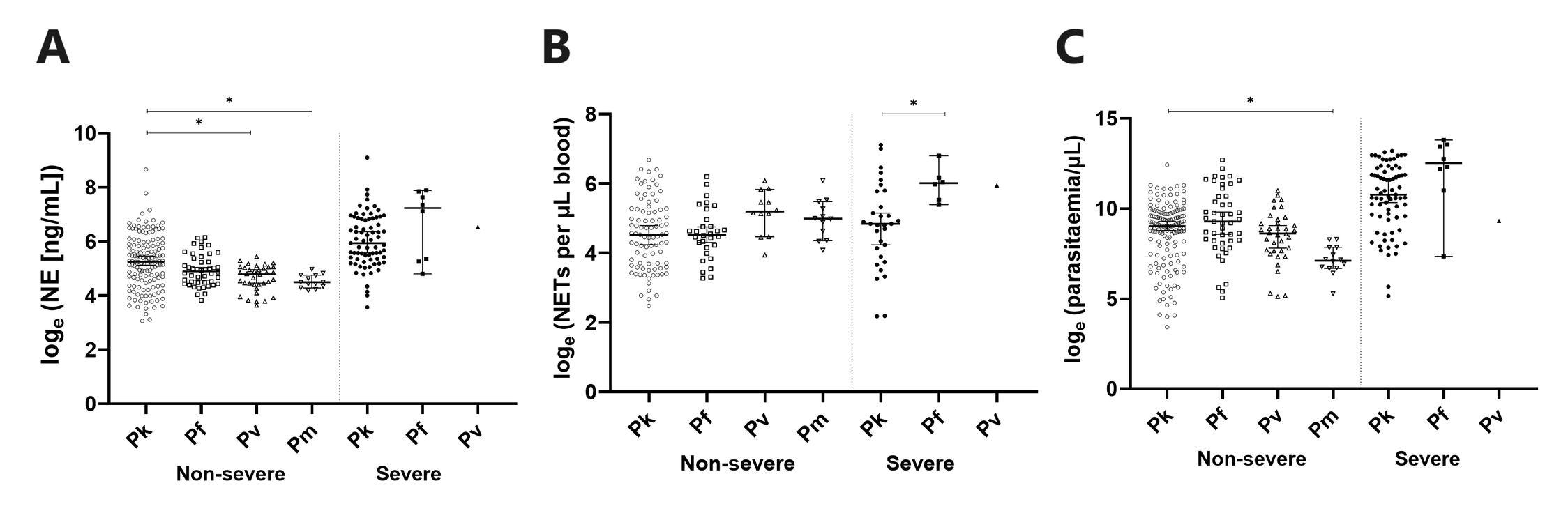

Supplement: S4 Fig — Comparison of (A) neutrophil elastase, (B) circulating neutrophil extracellular traps and (C) parasitaemia of P. knowlesi against P. falciparum, P. vivax and P. malariae (from Timika, Indonesia [15]) in severe and non-severe cohorts. (TIF) [file pntd.0012424.s006.tif]
